# Arginine Vasopressin Modulates Ion and Acid/Base Balance by Regulating Cell Numbers of Sodium Chloride Cotransporter and H^+^-ATPase Rich Ionocytes

**DOI:** 10.3390/ijms21113957

**Published:** 2020-05-31

**Authors:** Sok-Keng Tong, Hung-Ling Lee, Yi-Chun Lee, Liang-Chun Wu, Yi-Ling Tsou, Shao-Wei Lu, Shang-Wu Shih, Pung-Pung Hwang, Ming-Yi Chou

**Affiliations:** 1 Department of Life Science, National Taiwan University, Taipei 10617, Taiwan; sokkeng.tong@gmail.com (S.-K.T.); frank4xx36@gmail.com (S.-W.S.); 2 Institute of Cellular and Organismic Biology, Academia Sinica, Taipei 11529, Taiwan; lisali9975@gmail.com (H.-L.L.); K2105283@gate.sinica.edu.tw (Y.-C.L.); wulight0129@gmail.com (L.-C.W.); amydc10@gmail.com (Y.-L.T.); bluerclouder@gmail.com (S.-W.L.); pphwang@gate.sinica.edu.tw (P.-P.H.)

**Keywords:** vasopressin, ionocyte, and ion regulation

## Abstract

Arginine vasopressin (Avp) is a conserved pleiotropic hormone that is known to regulate both water reabsorption and ion balance; however, many of the mechanisms underlying its effects remain unclear. Here, we used zebrafish embryos to investigate how Avp modulates ion and acid–base homeostasis. After incubating embryos in double-deionized water for 24 h, *avp* mRNA expression levels were significantly upregulated. Knockdown of Avp protein expression by an antisense morpholino oligonucleotide (MO) reduced the expression of ionocyte-related genes and downregulated whole-body Cl^−^ content and H^+^ secretion, while Na^+^ and Ca^2+^ levels were not affected. Incubation of Avp antagonist SR49059 also downregulated the mRNA expression of sodium chloride cotransporter 2b (*ncc2b*), which is a transporter responsible for Cl^−^ uptake. Correspondingly, *avp* morphants showed lower NCC and H^+^-ATPase rich (HR) cell numbers, but Na^+^/K^+^-ATPase rich (NaR) cell numbers remained unchanged. *avp* MO also downregulated the numbers of *foxi3a*- and p63-expressing cells. Finally, the mRNA expression levels of *calcitonin gene-related peptide* (*cgrp*) and its receptor, *calcitonin receptor-like 1* (*crlr1*), were downregulated in *avp* morphants, suggesting that Avp might affect Cgrp and Crlr1 for modulating Cl^−^ balance. Together, our results reveal a molecular/cellular pathway through which Avp regulates ion and acid–base balance, providing new insights into its function.

## 1. Introduction

Arginine vasopressin (Avp), sometimes also called antidiuretic hormone, is synthesized by neurons in the mammalian anterior hypothalamus that project to the posterior pituitary. In response to stimuli, Avp is released from the posterior pituitary and enters the circulatory system [1]. Previous studies suggested that AVP participates in cation transport in the renal tubules, regulating the reabsorption of Na^+^, Mg^2+^, and Ca^2+^, as well as the secretion of K^+^ in the distal segment of the nephron; it also modulates intracellular pH in the collecting duct cells [2,3,4]. In mammals, Avp responds to sudden body fluid fluctuations by inducing posttranslational modifications and trafficking of the water channel, aquaporin-2 [5,6]. Avp also regulates NaCl reabsorption by phosphorylating the Na^+^-K^+^-2Cl^−^ cotransporter (Nkcc2) and Na^+^-Cl^−^ cotransporter (Ncc) in the thick ascending limb of Henle’s loop and distal convoluted tubule, respectively [7]. Notably, injection of synthetic Avp (deamino-Cys-1, d-Arg-8 vasopressin, dDAVP) into Avp-deficient transgenic rats was able to stimulate Ncc phosphorylation, which led to translocation of Ncc to the apical membrane [8]. 

As an important regulator of kidney function, Avp is considered to be a promising pharmacological target for autosomal dominant polycystic kidney disease (ADPKD), a common inherited kidney disorder. Patients with ADPKD develop cysts with abnormal fluid accumulation and exhibit increased cell mass in kidney tubules [9]. Increased plasma Avp is associated with an elevated cellular cAMP level in ADPKD patients, and this elevation becomes more pronounced as the disease progresses [10]. To counteract upregulation of cAMP and subsequent cellular over-proliferation, a vasopressin receptor 2 (V2R) antagonist may be administered to relieve cyst development. Indeed, reduced V2R signaling was found to partially rescue the excessive proliferation of collecting duct cells [11]. Thus, Avp appears to play an important role in epithelial cell proliferation of the collecting duct, which causes cyst formation in the kidney tubules. 

The Avp-mediated regulation of body fluid ionic homeostasis may have evolved in early vertebrates. Arginine vasotocin (Avt; current name: arginine vasopressin, Avp) is a homologous oligopeptide of Avp found in non-mammalian vertebrates and has been reported to regulate body fluid homeostasis in fishes and amphibians [12,13,14]. In medaka, rainbow trout, and flounder, pituitary storage of Avp is significantly decreased, and plasma Avp is increased after transferring the fishes to hyperosmotic environments [15,16,17,18,19]. Thus, Avp is likely to play an important role in fish salt excretion during hyper-osmoregulation in seawater. Avp was also reported to stimulate Na^+^ and Cl^−^ transport in abdominal skin of the Japanese Buerger’s frog and pond green frog, respectively [20]. Recently, Lema et al. observed that Avp possesses dual osmoregulatory roles in pupfish. As such, Avp diminishes mRNA expression of *ncc2* in the gills of fishes in hypoosmotic environments and stimulates cystic fibrosis transmembrane conductance regulator (*cftr*) mRNA expression in hyperosmotic environments. Thus, Avp serves to inhibit Cl^−^ uptake and facilitate Cl^−^ secretion during seawater acclimation [12]. Because Avp functions differently in different species, its complex effects on osmo/iono-regulation must be carefully delineated in each system, and as yet unidentified conserved regulatory modules are likely to exist. Moreover, the molecular physiological mechanisms of these regulatory pathways, including the target genes and cells, remain largely unknown and require further elucidation. 

Compared to mammals, zebrafish have a simpler body plan and similar organ systems. Furthermore, the functions of zebrafish genes and cells can be easily evaluated in vivo by various molecular physiological approaches. Thus, zebrafish have been firmly established as a competent model system for developmental biology, neurobiology, and physiology [21,22,23,24,25]. Ion and acid–base homeostatic mechanisms in zebrafish have been well studied and are analogous to those in mammalian distal convoluted tubules and collecting duct with regard to expression and function of ion transporters [22,26,27,28,29,30,31]. Since Avp regulates water and ion balance and also acts as a regulator for cell proliferation, it is possible that Avp affects body fluid ion and acid–base balance by regulating proliferation and/or differentiation of ion-transporting ionocytes. To test this hypothesis, we used zebrafish as a model to examine the effects of Avp on the expression of pertinent ion transporter genes, proliferation and differentiation of ionocyte progenitor cells, and ion transporter functions. Our findings describe the action of Avp on Cl^−^ uptake and acid/base regulation mechanisms, enhancing our understanding of vertebrate osmoregulation and providing insights into the comparative physiology of Avp.

## 2. Results

### 2.1. Avp and its Receptors are Widely Expressed in Zebrafish Tissues

**Figure 1:**
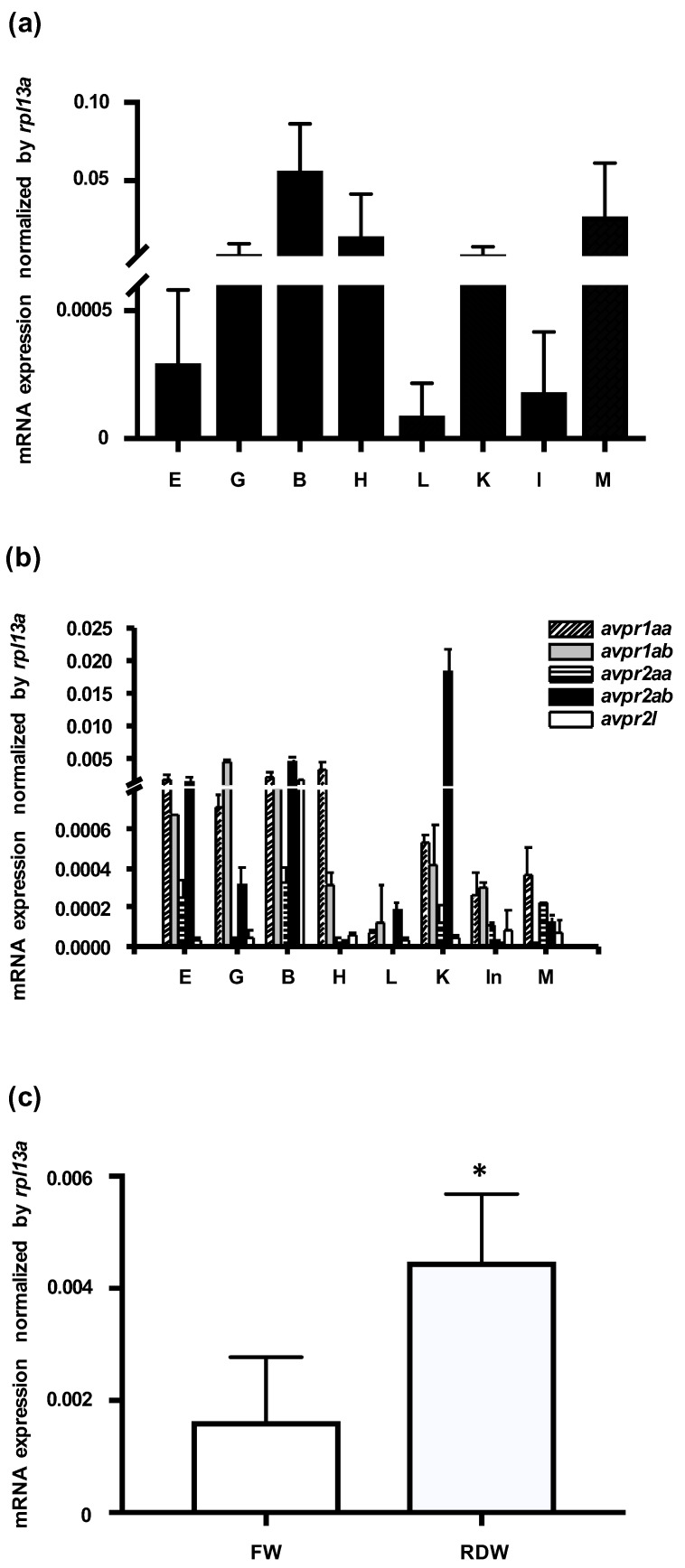
Expression levels of mRNAs encoding arginine vasopressin (Avp) and Avp receptors in zebrafish. Relative mRNA expression level of Avp (**a**) and five Avp receptors (**b**) in the eye (E), gill (G), brain (B), heart (H) liver (L), kidney (K), intestine (In), and muscle (M) of adult zebrafish. Values were normalized to *rpl13a*. Relative expression of *avp* in 3-days post-fertilization (dpf) embryos grown (**c**) in freshwater (FW) or RO-deionized water (RDW) for 24 h. Quantification was performed by standard qRT-PCR methods. Values are mean ± s.e.m. (*n* = 4–5). Asterisk indicates significant difference between the two groups, according to Student’s *t*-test, *p* < 0.05.

mRNA expression of zebrafish *avp* and its five receptors (*avpr1aa*, *avpr1ab*, *avpr2aa*, *avpr2ab*, and *avpr2l*; accession numbers: NM_001301114, NM_001297676, XM_009296925, XM_001922007, and NM_001110125, respectively) was examined in various tissues by qRT-PCR, using *rpl13a* as an internal control. *avp* mRNA was abundantly expressed in brain, muscle, heart, kidney, and gill, and it was moderately expressed in eye, liver, and intestine (Figure 1a). Five Avp receptors were also differentially expressed in the examined tissues. Expression levels were highest for *avpr1aa* in heart, for *avpr1a* in gill, for *avpr2aa* and *avpr2l* in brain, and for *avpr2ab* in kidney (Figure 1b). 

To examine how environmental salinity affects *avp* expression, we applied a hypotonic stimulus to zebrafish embryos. At 72 h post fertilization (hpf), embryos were transferred from freshwater to RO-deionized water for 24 h, and *avp* expression levels were measured by qRT-PCR. As shown in Figure 1c, *avp* mRNA was significantly upregulated after deionized water treatment. 

### 2.2. avp Knockdown Downregulates Avp Protein Expression

**Figure 2:**
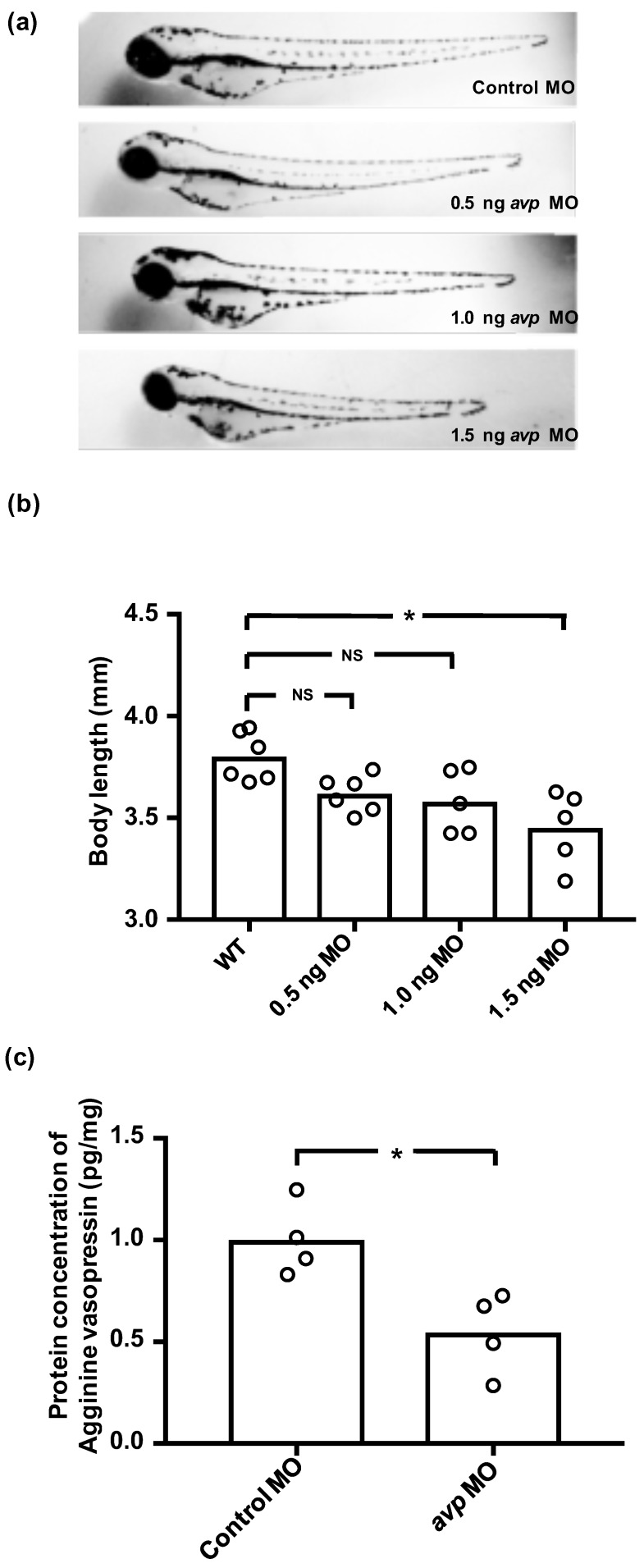
Effects of downregulating Avp by morpholino injection in zebrafish embryos. (**a**) Comparison of morphology and body length at 3 dpf for uninjected wild-type larvae (WT) and those injected with various doses of *Avp* morpholino (MO) at 1–2-cell stage. (**b**) Histogram shows body length with individual measurements indicated by open circles. (**c**) Relative protein level of AVP at 3 dpf was assessed by enzyme-linked immunosorbent assay (ELISA) in larvae injected with 1 ng of control morpholino or *Avp* morpholino (AVP MO). Asterisk indicates a significant difference from uninjected wild type (**b**) or control (**c**), according to one-way ANOVA, Tukey’s pair-wise comparison, and Student’s *t*-test, *p* < 0.05. N.S. indicates not significant.

To investigate the functional role of Avp in ion regulation, we used *avp* morpholino (MO) to interfere with Avp synthesis. We tried three dosages of *avp* MO (0.5, 1, and 1.5 ng/embryo), and found the high dose (1.5 ng) caused a decrease in body length (Figure 2a,b). In contrast, morphants injected with 0.5 or 1 ng of *avp* MO appeared similar to control MO-injected embryos; mortality, hatching rate, body length, and body shape were all similar between the lower-dose morphants and control MO-injected embryos at 72 hpf (Figure 2a,b). Therefore, we chose 1 ng/embryo as a standard dose for subsequent experiments. Using this dosage, we were able to minimize developmental perturbations induced by the *avp* MO, while retaining the effects of *avp* depletion on physiological responses. 

To test the efficiency and specificity of the *avp* MO, we measured Avp protein concentrations in morphants and control MO-injected embryos by enzyme-linked immunosorbent assay (ELISA). At 72 hpf, Avp protein levels in morphants were significantly lower than those in control MO-injected embryos, suggesting that *avp* MO efficiently blocked Avp protein synthesis (Figure 2c). 

### 2.3. Avp Regulates the Function of Ionocytes

**Figure 3:**
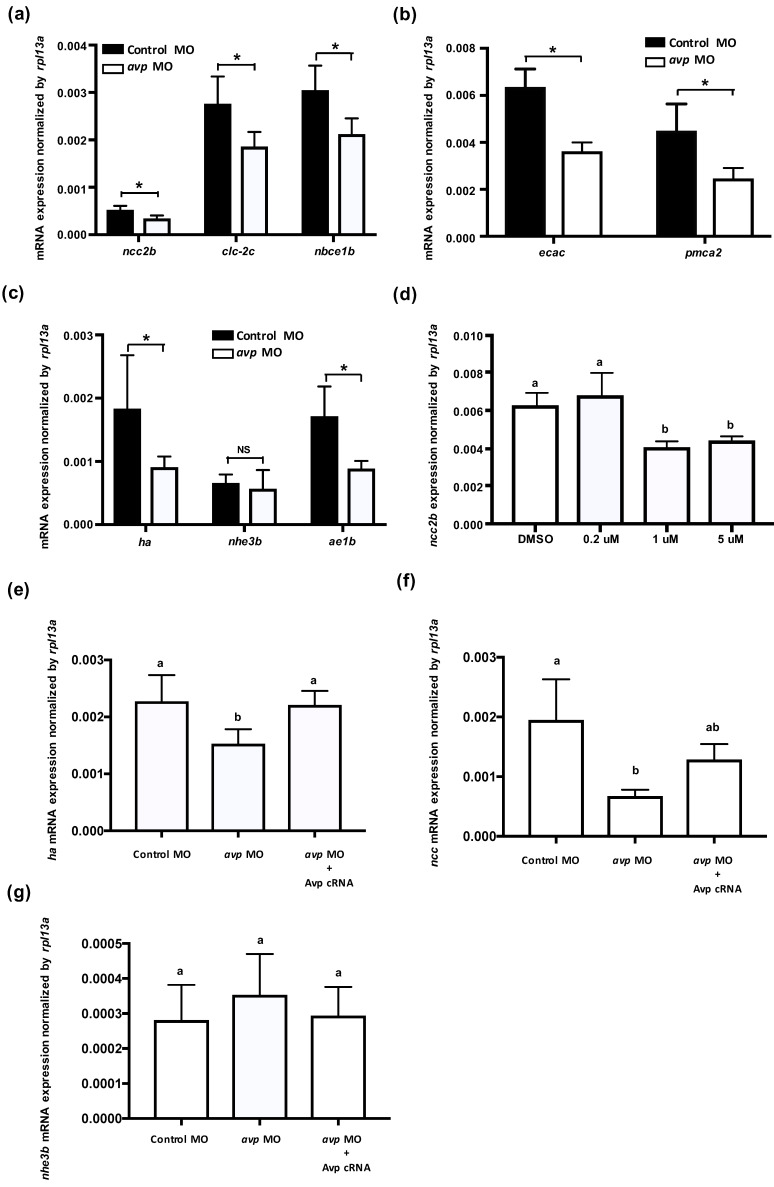
Effects of morpholino-mediated downregulation of Avp on the mRNA expression of ionocyte-related genes. Comparison of mRNA levels encoding ion transporters in 3-dpf-lavae injected with control morpholino (black bar) and *avp* morpholino (white bar); genes for NCC2b, CLC-2c, and NBCe1b were expressed in NCC cells (**a**); ECaC and PMCA2 were expressed in Na+/K+-ATPase rich (NaR) cells (**b**); HA, NHE3b, and AE1b were expressed in NCC and H+-ATPase rich (HR) cells (**c**). The NCC2b expressions were significantly downregulated by antagonist SR49059 treatments (1 and 5 μM) (**d**). Co-injection of *avp* MO and Avp cRNA rescued the mRNA expressions of *ha* (**e**) and *ncc2b* (**f**), while the *nhe3b* mRNA expression was not affected by *avp* MO and Avp cRNA (**g**). Bracket labeled with NS indicates no significant difference from control MO-injected samples. Values are mean ± s.e.m. (*n* = 4–5). Brackets with asterisks indicate a significant difference from control, according to Student’s *t*-test, *p* < 0.05. Different letters indicate a significant difference between each treatment, according to one-way ANOVA, Tukey’s pair-wise comparison, *p* < 0.05.

In zebrafish, at least three types of ionocytes were identified in the skin/gills: Na^+^-K^+^-ATPase-rich cell (NaRC), H^+^-ATPase-rich cell (HRC), and Na^+^-Cl^−^ cotransporter cell (NCC), which are responsible for calcium uptake, acid/base balance, and chloride uptake, respectively [28,32,33]. mRNA expression levels of ion transporters that are found in NCC cells (*ncc2b*, *clc-2c*, and *nbce1b*) were significantly downregulated by *avp* MO (Figure 3a); gene expression of the NaR cell-related genes (*ecac* and *pmca2*) was also downregulated by *avp* MO (Figure 3b). Among HR cell-related genes, mRNA expression of *ha* and *ae1b* were decreased significantly in the morphants, while *nhe3b* expression did not change compared with control MO-injected embryos (Figure 3c). To further test the effects of Avp on mRNA expression of ionocyte-related genes, we incubated the embryos with Avp antagonist SR49059 (for receptor type1A), SSR149415 (for receptor type 1B), and Tolvaptan (for receptor type2) and checked the mRNA expression of *ncc2b*. The *ncc2b* expressions were significantly downregulated by antagonist SR49059 treatments (1 and 5 μM) (Figure 3d), while SSR149415 and Tolvaptan did not significantly affect *ncc2b* expressions (data not shown). In addition, we co-injected *avp* MO with Avp cRNA and found that Avp cRNA rescued the mRNA expressions of *ha* (Figure 3e) and *ncc2b* (Figure 3f), while the nhe3b mRNA expression was not affected by *avp* MO and Avp cRNA (Figure 3g).

**Figure 4:**
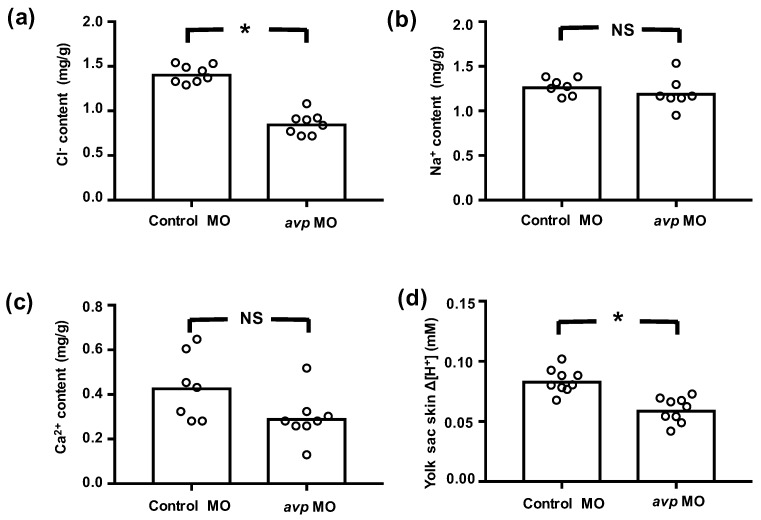
Effects of downregulating Avp on ion content or efflux at 3 dpf. Embryos were injected with control morpholino (Control MO) or antisense *avp* morpholino (*avp* MO) at the 1–2-cell stage. Mean values for whole body Cl^−^ (**a**), Na^+^ (**b**), and Ca^2+^ (**c**) contents of 8 samples (10 embryos each) are shown in the histograms, with individual values indicated by white circles. Cl^−^ content was determined by spectrophotometer at 460 nm upon reaction with mercury thiocyanate and iron alum. Na^+^ and Ca^2+^ contents were measured by atomic absorption spectrophotometry. H^+^ flux gradients (**d**) between skin surface on yolk sac and nearby buffer (by a 10 mm-distance horizontally) were determined by the scanning ion-selective electrode technique (SIET). A positive H^+^ gradient (Δ[H^+^]) representing acid secretion was recorded at the surface of the yolk sac. Values are means (*n* = 8–9). Asterisks indicate significant differences from control, according to Student’s *t*-test, *p* < 0.05. NS indicates no significant difference.

We further measured whole body contents of Cl^−^, Ca^2+^, and Na^+^, as well as the secretion of H^+^, to evaluate the functional effects of *avp* MO on ionocytes. In *avp* morphants, whole body Cl^−^ content was significantly lower than that in control MO-injected embryos, but Ca^2+^ and Na^+^ contents were not different (Figure 4). Morphants also showed lower levels of H^+^ secretion (Figure 4). Together, these results suggest that Avp regulates the function of NCC and HR cells, with minor effects on the function of NaR cells. 

### 2.4. Avp Modulates the Number of Ionocytes on the Skin

**Figure 5:**
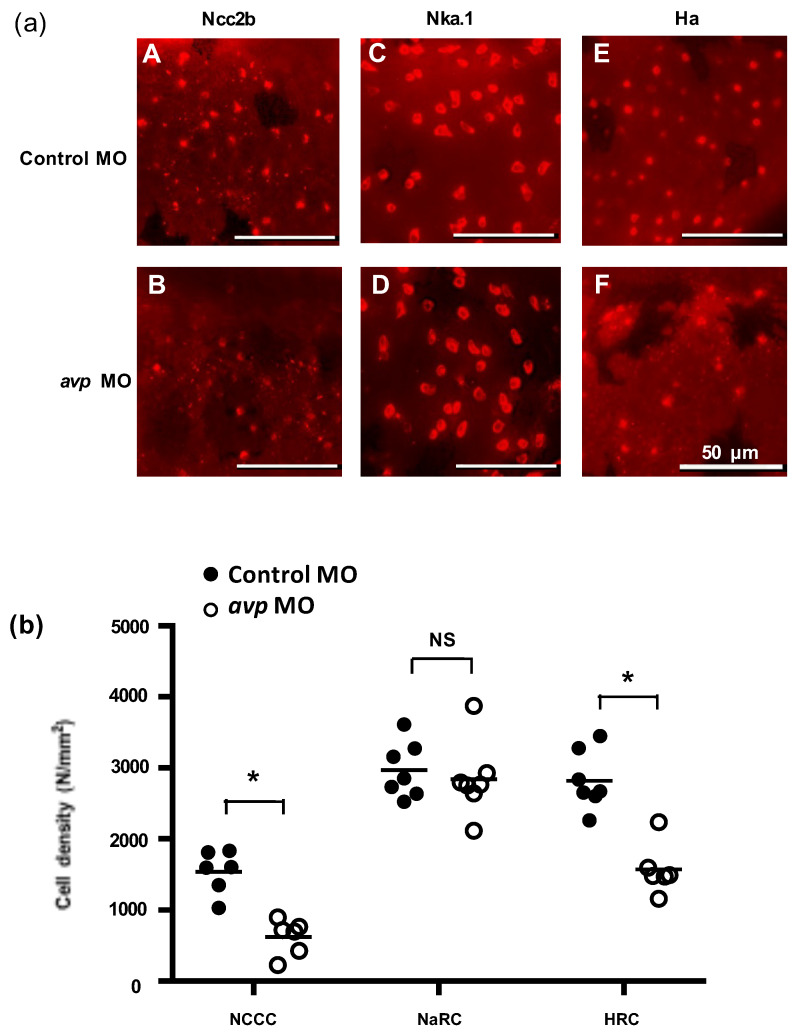
Effects of morpholino-mediated downregulation of AVP on ionocyte cell density in 3 dpf embryos. (**a**) NCC (A,B), NaR (C,D), and HR (E,F) cells were visualized by immunofluorescence labeling, using antibodies against NCC2b (A,B), NKA.1 (C,D), and HA (E,F), respectively at 3 dpf. Scale bars represent 50 μm. Mean cell density of ionocytes (**b**) is shown as a solid line with individual values indicated by black-filled circles for control morpholino-injected larvae or white-filled circles for antisense *Avp* mopholino-injected larvae. Values are means (*n* = 6–8). Asterisks indicate significant difference from control, according to Student’s *t*-test, *p* < 0.05. NS indicates no significant difference.

**Figure 6:**
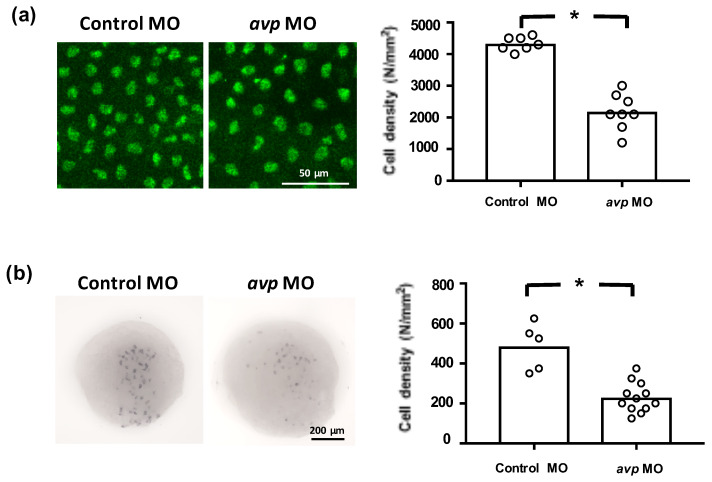
Effects of morpholino-mediated downregulation of AVP on ionocyte precursor cell density. Epithelial stem cells at 3 dpf (**a**) and ionocyte progenitors at the tail-bud stage (**b**) were labeled by p63 antibody and *foxi3a* antisense riboprobe, respectively. Mean cell densities of p63- or *foxi3a*-expressing cells are indicated by bars with individual values shown as white-filled circles. Values are means (*n* = 5–12). Asterisks indicate a significant difference from control group, according to Student’s *t*-test, *p* < 0.05.

To investigate whether Avp is involved in the development of zebrafish ionocytes (the major ion-regulating cells), we knocked down Avp protein expression and observed the effects on NCC, NaR, and HR cell populations at 72 hpf. The *avp* MO diminished the numbers of NCC and HR cells but did not change the number of NaR cells on zebrafish embryo skin (Figure 5). We next examined if Avp affects cell number and differentiation of ionocyte progenitor cells. We performed RNA in situ hybridization to detect helix/forkhead box transcription factor, *foxi3a*, which encodes the master regulator of ionocyte differentiation in zebrafish at the tail-bud stage [34]. At this stage, zebrafish embryos express *foxi3a* but do not express any ion transporter genes. We found that the number of cells expressing *foxi3a* was decreased by *avp* MO (Figure 6), indicating that it regulates the populations of ionocyte progenitor cells. Moreover, we performed immunofluorescence for p63, a marker of epithelial stem cells [35], at 72 hpf. The density of p63-positive cells was significantly lower in Avp morphants than in control MO-injected embryos (Figure 6). 

### 2.5. Avp Morphants Exhibit Reduced Expressions of Cgrp and its Receptor Crlr1

**Figure 7:**
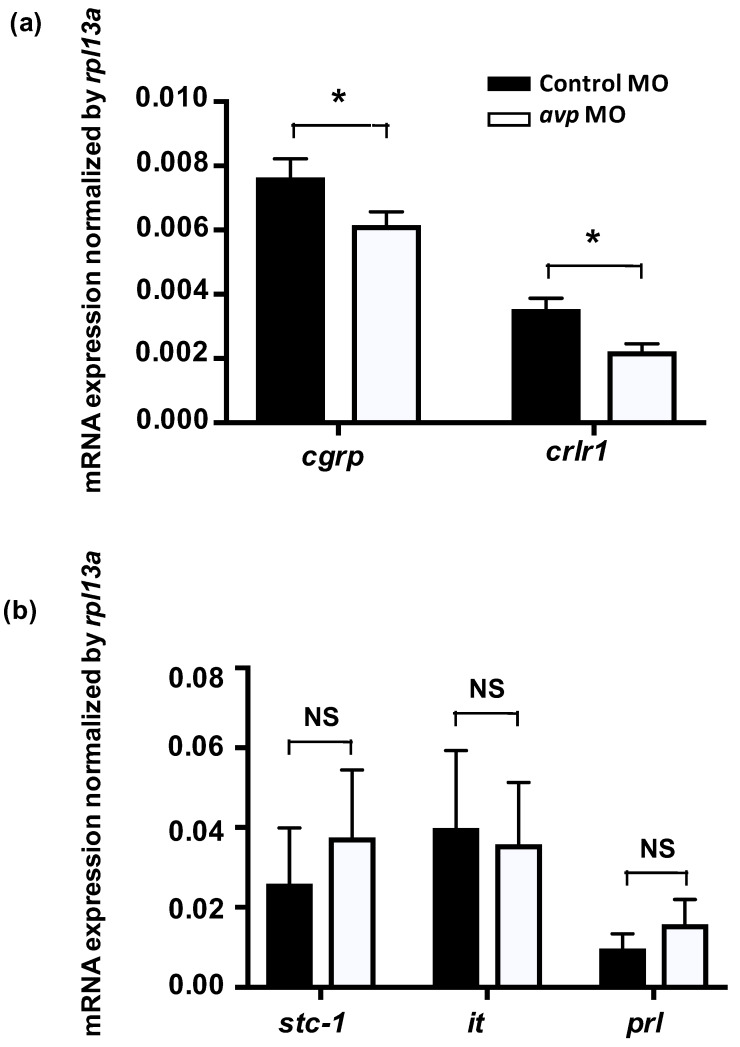
Effects of morpholino-mediated AVP downregulation on mRNA expression levels of hormone-related genes at 3 dpf, as measured by qRT-PCR. Relative expression levels of *calcitonin gene-related peptide* (*cgrp*) and its receptor (*crlr1*) in fish injected with control morpholino (black bar) or antisense *avp* morpholino (white bar) are shown (**a**). Both genes exhibited significant differences between control and *avp* morpholino-injected samples. Relative expression levels of hormone-related genes in fish injected with control morpholino (black bar) or antisense *avp* morpholino (white bar) are shown (**b**). The examined genes include *stanniocalcin-1* (*stc-1*), *isotocin* (*it*), and *prolactin* (*prl*). Values are mean ± s.e.m. (*n* = 4–5). Asterisks indicate significant difference between control and *Avp* morpholino-injected samples, according to Student’s *t*-test, *p* < 0.05. NS indicates no significant difference.

To investigate if Avp functions with other hormones to modulate Cl^−^ balance, we knocked down Avp protein expression and measured expression of genes for *calcitonin gene-related peptide* (*cgrp*), *calcitonin receptor-like 1* (*crlr1*), *stanniocalcin-1* (*stc-1*), *isotocin*, and *prolactin*, which have all been reported to participate in Cl^−^ balance in zebrafish [36,37,38,39]. The expression levels of mRNA encoding *cgrp* and its receptor *crlr1* were downregulated in Avp morphants (Figure 7). In contrast, expression of genes encoding Stc-1, isotocin and prolactin were not affected by *avp* MO (Figure 7). 

## 3. Discussion

Our study shows that Avp participates in ion regulation and is especially important for Cl^−^ balance and acid/base balance. Avp increases cell number of epithelial stem cells and ionocyte progenitor cells, and these actions likely affect the densities of NCC and HR cells to respectively modulate Cl^−^ uptake and acid secretion functions in zebrafish. These results provide molecular physiological evidence of a novel action of Avp in regulating body fluid ion and acid–base homeostasis in fishes. 

In addition to the expression in the brain, *avp* expression has also been reported in the bone marrow and testes [40,41], suggesting it has both central and peripheral effects. In the present study, zebrafish *avp* showed ubiquitous expression, but it was most highly expressed in the brain (Figure 1a). This result is in agreement with the fact that Avp is known to be mainly produced by the hypothalamic neurons. The wide distribution pattern of *avp* expression in many tissues, including the eyes, gills, heart, kidney, and muscle, is similar to distributions found in Japanese quail, chicken, lamprey, and shark [42,43,44,45]. This wide distribution is also in line with the idea that Avp may regulate a variety of physiological functions, such as muscle contraction, reproduction, salt acclimation, and social behavior [12,46,47,48,49]. Furthermore, we found that five Avp receptors were widely expressed in various tissues (Figure 1b). The broad distribution of Avp and its receptors in mammalian and non-mammalian tissues suggests that multiple functions of Avp are conserved among vertebrates. 

Regarding its role in ion regulation, Avp was shown to be involved in Ncc and Nkcc-mediated Cl^−^ uptake in mouse medullary thick ascending limb (TAL) [50], and an increase in circulating Avp was associated with a higher protein level of apical Nkcc in rat TAL [51]. In a chronic (7-day) study, the infusion of dDAVP increased the Ncc protein level [52]. On the other hand, Avp has also been found to participate in acid–base regulation. Avp increased the abundance of apical vacuolar H^+^-ATPase in intercalated cells, an action that contributes to urinary acidification [53,54]. Correspondingly, defects in the Avp V1a receptor in intercalated cells caused renal tubular acidosis [55]. In teleost fishes, Avp also participates in osmo/iono-regulation. Several studies in different species showed that the release of Avp from the pituitary is increased during seawater acclimation [15,16,17,18,19], while hypotonic stimulation also induces Avp secretion in both flounder and trout [56]. Although Avp has been proposed to regulate Nkcc2 in the intestine of sea bream [57] and stimulate Na^+^ and Cl^−^ transport in the skin of frog [20], the details of these regulatory mechanisms, such as the target molecules and cells/tissues that respond to Avp, are still not clear and need further elucidation. Since several studies in fishes have shown that Avp is involved in seawater acclimation [12,15,16,17,18,19], our results demonstrate a complementary role of Avp in acclimation to a hypotonic environment (Figure 1c). We also found that Avp is important for Cl^−^ and H^+^ balance but plays only a minor role at most in Ca^2+^ and Na^+^ regulation (Figure 4 and Figure 5). Thus, we conclude that gill/skin ionocytes are major target cells of Avp, and Avp acts as a positive regulator for body fluid Cl^−^ and acid–base homeostasis, probably by affecting proliferation and differentiation of ionocytes.

Since *avp* MO downregulated densities of certain ionocytes (Figure 5), it is possible that Avp specifically regulates the proliferation and differentiation of ionocyte progenitor cells. Several studies have previously shown that Avp acts as a mitogen and growth-promoting factor to modulate cell proliferation and differentiation. For example, Avp induces the production of cardiac fibroblasts [58,59] and stimulates neonatal rat cardiac fibroblasts through vasopressin receptor type1A signaling [60]. In embryonic stem cells, Avp promotes cardiomyocyte differentiation by elevating the mRNA and protein levels of endothelial nitric oxide synthase and prolonging action potentials in ventricular cells [61]. Moreover, Avp stimulates mesangial cell growth by activating both the phosphatidylinositol 3-kinase and Ras-mitogen-activated protein kinase pathways [62]. In teleost fishes, the mitogenic effects of Avp are poorly understood. To test whether Avp affects proliferation and differentiation of ionocytes, we compared cell densities of *foxi3a*- and p63-expressing cells in the skin of Avp morphants and control MO-injected embryos. Zebrafish Foxi3a is widely regarded as a mater regulator of ionocyte differentiation [34,63], and p63 is a marker of epidermal stem cells, which are the cellular source of ionocytes and keratinocyte progenitors [64]. Our results showed that *avp* MO downregulated cell densities of both *foxi3a*- and p63-expressing cells (Figure 6); this modulation of progenitors may then influence numbers of ionocytes to regulate Cl^−^ and H^+^ homeostasis. It should be noted that the exclusion of ionocyte cell numbers modulated by apoptosis requires further experiments.

Notably, the effects of Avp loss-of-function on Ca^2+^ uptake showed inconsistencies in terms of whole-body Ca^2+^ content, differentiation of the ionocytes, and expression of transporters related to Ca^2+^ uptake function (Figure 3, Figure 4 and Figure 5). Since Avp appears to exert minor or insignificant effects on the differentiation of Ca^2+^-transporting ionocytes (NaR cells, Figure 3), the changes in transporter expression may reflect an interplay between different hormones or the secondary effects of internal acidosis. We observed that *avp* MO impaired acid secretion function (Figure 3, Figure 4 and Figure 5), and subsequent internal acidosis might have suppressed Ca^2+^ uptake mechanisms in the morphants, similar to what was previously reported in zebrafish with knockdown of Gcm2 (a specific transcription factor for HR cells) [65] or H^+^-ATPase [66] and in mammals with internal acidosis syndrome [67,68].

Control of body fluid homeostasis in organisms requires the coordinated participation and interaction of many hormones that simultaneously act at different functional levels of ion transport. Several hormones, including aldosterone, angiotensin II, prolactin, and Avp, are known to regulate Ncc function in mammalian kidneys [69]. However, the specific signals and pathways that govern Ncc expression and function are still unknown. In mammals, Avp is known to participate with serotonin, epinephrine, aldosterone, angiotensin, corticotropin-releasing hormone, and oxytocin to modulate behavior, blood pressure, acid–base balance, urine production, stress response, and ion excretion [55,70,71,72,73,74,75]. In teleost fishes, Avp was proposed to interact with isotocin, melatonin, and urotensin II to respectively regulate plasma cortisol concentration, blood pressure, and release of isotocin [76,77,78]. Several hormones (i.e., stanniocalcin-1, isotocin, prolactin, cortisol, estrogen-related receptor, endothelin, and Cgrp) have been demonstrated to regulate Cl^−^ uptake and acid secretion in zebrafish [22,36,37,38,39]. Therefore, we evaluated the possible interplay of these hormonal factors with Avp by examining the effects of *avp* MO on gene expression of the hormones, their receptors, and their synthesis enzymes. Most of the hormone-related genes exhibited non-significant or inconsistent changes, and accordingly, their crosstalk with Avp may be limited or in need of clarification by further studies. Only *cgrp* and *crlr1* expression levels were consistently downregulated by *avp* MO, (Figure 7), suggesting that Avp is likely to influence the Cgrp/Crlr pathway to specifically regulate Cl^−^ uptake. A previous study showed that Cgrp inhibits bicarbonate secretion in the rat duodenum by activating the sympathetic efferent and subsequently stimulating the release of norepinephrine and Avp [79]. In zebrafish, our previous study demonstrated that Cgrp-Crlr1 functions as a negative regulatory axis, which suppresses *ncc2b* expression to diminish Cl^−^ uptake [38]. In the present study, Avp functionally stimulated Cl^−^ uptake, probably via the upregulation of *ncc2b* expression. To compensate the impairments in Cl^−^ homeostasis in *avp* morphants, including the reduction in *ncc2b* expression (Figure 3a), the lower number of NCC cells (Figure 4), and diminished whole-body Cl^−^ content (Figure 5), zebrafish appear to limit the expression of genes for Cgrp and Crlr1, which would disinhibit *ncc2b* expression. Therefore, crosstalk and balance between Avp and Cgrp signals may be important in the regulation of Cl^−^ uptake.

The positive regulatory roles of Avp on body fluid Cl^−^ and acid–base homeostasis that we observed in zebrafish are in accordance with its known physiological functions in mammalian kidneys. The present study further reveals novel effects of Avp on Ncc2b and H^+^-ATPase expression at mRNA and protein levels. For the first time, we demonstrated that Avp modulates cell number of epithelial stem cells and the ionocyte progenitor cells to control the number of NCC and HR cells and regulate Cl^−^ uptake and acid secretion. Moreover, this regulatory mechanism may be mediated by crosstalk between Avp and Cgrp–Crlr1 signaling. Interestingly, the skin/gill ionocytes in zebrafish and the collecting duct intercalated cells in mammals show similar cell differentiation pathways, which involve similar signaling components (i.e., Fox and Notch) [34,80]. The present findings on zebrafish Avp provide a rationale to examine further whether Avp also controls epithelial Cl^−^ uptake and acid secretion functions through the regulation of cell differentiation in the mammalian kidney. Thus, our findings provide new insights into hormonal control of body fluid Cl^−^ and acid–base homeostasis, adding to our understanding of vertebrate endocrinology.

## 4. Materials and Methods

### 4.1. Animals

AB strain zebrafish were obtained from the Institute of Cellular and Organismic Biology, Academia Sinica. Fish were kept in local tap water with a circulation system at 28.5 °C under a 14:10-h light–dark photoperiod. The experimental protocols were approved by the Academia Sinica Institutional Animal Care and Utilization Committee (approval no. RFIZOOHP220782).

### 4.2. Preparation of Total RNA and cDNA Synthesis

To obtain sufficient RNA, 30 embryos were pooled for each sample. Embryos were homogenized in 0.8 mL Trizol reagent (Invitrogen, Carlsbad, CA, USA). After chloroform extraction, total RNA was purified and treated with DNase I to remove genomic DNA by an RNeasy Mini Kit (Qiagen, Hilden, Germany). For cDNA synthesis, 5 μg total RNA was reverse-transcribed at 42 °C for 30 min in a final volume of 20 μL [0.5 mM dNTPs, 2.5 μM oligo(dT)18, 5 mM dithiothreitol, and 200 units PowerScript reverse transcriptase (Invitrogen)], followed by incubation at 70 °C for 15 min.

### 4.3. Quantitative Real-Time Polymerase Chain Reaction (qRT-PCR)

qRT-PCR was performed with a LightCycler real-time PCR system (Roche Applied Science, Penzberg, Germany) in a final volume of 10 μL [5 μL 2x SYBR green I Master (Roche, Basel, Switzerland), 300 nM of primer pairs, and 20–30 ng cDNA]. The gene encoding ribosomal protein L13a (*rpl13a*; ENSDARG00000044093) was used as an internal control. For each gene, a standard curve was used to confirm signals were in the linear range. The specificity of the primer sets was validated by the presence of a single peak in the dissociation curve. Primer sets used for RT-qPCR are shown in Table 1.

### 4.4. Microinjection of Avp Antisense Morpholino Oligonucleotide (MO) and Capped-mRNA (cRNA)

The *avp* morpholino oligonucleotide (MO) and mismatched-*Avp* MO were obtained from Gene Tools (Philomath, OR, USA). The sequences of MO were as follows: *Avp* MO, 5′-AGACAGCAGAGAGTCTGACATCTCG-3′; *Avp*-mismatched MO, 5′-AGACACCAGACAGTGTCACTTCTCG-3′. The MOs were prepared with 1× Danieau solution [58 mM NaCl, 0.7 mM KCl, 0.4 mM MgSO_4_, 0.6 mM Ca(NO_3_)_2_, and 5.0 mM HEPES; pH 7.6]. MO solution containing 0.1% phenol red (for visualization) was injected into zebrafish embryos at the 1–2-cell stage using an IM-300 microinjector system (Narishigi Scientific Instrument Laboratory, Tokyo, Japan). For capped-mRNA (cRNA) injection, the corresponding Avp coding region was amplified by PRC and inserted into the pCS2^+^ vector. The construct was linearized with *Not*I, and cRNA was transcribed using an SP6 message RNA polymerase kit (Ambion, Huntington, UK). cRNA was injected into embryos at the 1- to 2-cell stage at 400 pg/embryo. 

### 4.5. Incubation of AVP Antagonists

To administer embryo with Avp antagonists, chorions were removed by 1 mg/mL pronase E (Roche) at 4 hpf followed immediately by different doses of antagonist treatment in freshwater. Thirty embryos were grouped in one well of a 12-well dish, and 6 wells were prepared for each treatment. Avp antagonist SR49059 (for receptor type1A, 2310 Tocris Bioscience, Bristol, UK), SSR149415 (for receptor type1B, 6195 Tocris), and Tolvaptan (for receptor type2, T7455, Sigma merged by Merck KGaA, Darmstadt, Germany) were dissolved in DMSO (0.2, 1, and 5mM) and added into freshwater at a final concentration of 0.2, 1, and 5 μM with DMSO maintained at 0.1%. We used the freshwater containing 0.1% DMSO as a control. Antagonist containing water was prepared fresh and renewed every day. Embryos were harvested at 3 days post-fertilization (dpf) for qRT-PCR.

### 4.6. Enzyme-Linked Immunosorbent Assay (ELISA) 

Twenty-five embryos were pooled in each sample to obtain sufficient protein for the assay. Isolated samples were homogenized in homogenization buffer (100 mM imidazole, 5 mM EDTA, 200 mM sucrose, and 0.1% sodium deoxycholate; pH 7.6), and then centrifuged at 4 °C and 10,000 rpm for 10 min. The protein levels were measured by an Arg^8^-Vasopressin ELISA Kit (Assay Designs, Ann Arbor, MI, USA). Samples containing 150 μg protein were loaded into each well and incubated at 4 °C for 24 h. The wells were then emptied and washed with a wash solution for three times. After the final wash, the trace wash buffer was removed, and the pNpp substrate solution was added to each well. Absorbance was measured at 450 nm in a synergy multi-mode plate reader (BioTek Instruments, Winooski, VT, USA) after stopping the enzymatic reaction with stop solution. The standard curve for AVP was constructed using commercial software (BioTek), and the concentration of AVP in unknown samples was determined by interpolation.

### 4.7. Measurement of Whole-Body Na^+^, Ca^2+^ and Cl^−^ Contents

Ten zebrafish embryos were rinsed briefly in deionized water and then pooled as one sample. HNO_3_ at 13.1 N was added to the samples for digestion at 60 °C overnight. Digested solutions were diluted with double-deionized water, and the total Na^+^ and Ca^2+^ contents were measured by atomic absorption spectrophotometry (Z-8000; Hitachi, Tokyo, Japan). For the measurement of Cl^−^ content, samples were homogenized with 1 mL deionized water and centrifuged at 14,000 rpm for 30 min. The supernatant was collected, followed by the addition of Hg(SCN_4_)_2_ (0.3 g in 95% ethanol) and NH_4_Fe(SO_4_)_2_·12 H_2_O (30 g in 135 mL 6 N HNO_3_) solutions for analysis. The Cl^−^ concentration was measured by the ferricyanide method with a double-beam spectrophotometer (model U-2000; Hitachi). Standard solutions of Na^+^, Ca^2+^, and Cl^−^ purchased from Merck (Darmstadt, Germany) were used to generate standard curves. 

### 4.8. Scanning Ion-Selective Electrode Technique (SIET) and Measurement of H^+^ Gradients

SIET was performed at room temperature (26–28 °C) in a small plastic recording chamber filled with 1 mL “recording medium”, which contained 300 µM 3-(N-morpholino)propanesulfonic acid (MOPS) buffer (Sigma), and 0.1 mg/L ethyl 3-aminobenzoate (Tricaine, pH 7.0) (Sigma) as described previously [32]. Anesthetized embryos were positioned in the center of the chamber with the lateral side contacting the base of the chamber. To record the H^+^ gradients at the surface of an embryo, the microelectrode was moved to a target position 10–20 µm away from the skin. After recording the target point, the microelectrode was then moved away (10 μm) to record the background. In the present study, ∆[H^+^] represents the measured H^+^ gradient between the point of interest (skin surface) and background.

### 4.9. Immunofluorescence (IF) and Cell Counting

Three days post-fertilization (dpf) zebrafish larvae were collected and fixed in 4% paraformaldehyde in phosphate-buffered saline (PBS) at 4 °C for 2 h. After being washed with phosphate buffered saline with tween 20 (PBST), samples were incubated with 3% bovine serum albumin (BSA) at room temperature for 2 h to prevent nonspecific binding. Primary antibodies included: anti-α5 monoclonal mouse antibody against avian Na^+^/K^+^-ATPase (1:200, Developmental Studies Hybridoma Bank, University of Iowa, Ames, IA, USA), anti-H^+^-ATPase rabbit polyclonal antibody against the A-subunit of zebrafish H^+^-ATPase (1:200; synthetic peptide: AEMPADSGYPAYLGARLA), anti-sodium chloride cotransporter 2b rabbit polyclonal antibody against the N-terminal domain (IKKSRPSLDVLRNPPDD) of zebrafish sodium chloride cotransporter 2b (1:100; customization produced by Genomics, Taipei, Taiwan) [38], and anti-P63 monoclonal mouse antibody against human P63 (1: 100; Santa Cruz Biotechnology, Santa Cruz, CA, USA). Signals were visualized with anti-rabbit or anti-mouse IgG conjugated with AlexaFluor 488 or 647 (1:200; Invitrogen). Images were acquired with a Leica TCS-SP5 confocal laser scanning microscope (Leica Lasertechnik, Heidelberg, Germany). For comparison of cell densities, NaR-, HR-, NCC-, and p63-expressing cells in 10–12 unit areas (100 × 100 μm) from one side of the yolk sac were counted and averaged for each individual.

### 4.10. In Situ Hybridization

A 750 bp fragment of the *forkhead box I3a* (*foxi3a*) gene was obtained by PCR and inserted into the pGEM-T easy vector (Promega, Madison, WI, USA). The inserted fragment was amplified with the M13 forward and M13 reverse primers by PCR, and the product was used as the template for in vitro transcription with SP6 and T7 RNA polymerase (Roche) in the presence of digoxigenin (DIG)-UTP (Roche) to synthesize the sense and antisense probes, respectively. DIG-labeled RNA probes were examined by using RNA gels to confirm their qualities. Zebrafish embryos were collected and fixed in 4% paraformaldehyde in phosphate-buffered saline (PBS) at 4 °C overnight. The samples were then washed by diethylpyrocarbonate (DEPC)-treated PBST (PBS with 0.1% Tween-20) five times (for 15 min each). After PBST washing, the samples were incubated with hybridization buffer (HyB: 50% formamide, 5× saline-sodium citrate (SSC) and 0.1% Tween 20) at 65 °C for 5 min and with HyB containing 500 μg/mL yeast tRNA at 65 °C for 4 h before hybridization. After overnight hybridization with 100 ng/mL DIG-labeled antisense or sense RNA probes, the embryos were serially washed with 50% formamide-2× SSC (at 65 °C for 20 min), 2× SSC (at 65 °C for 10 min), 2× SSC (at 65 °C for 10 min), 0.2× SSC (at 65 °C for 30 min, two times), and PBST at room temperature for 10 min. The embryos were then immunoreacted with an alkaline phosphatase-coupled anti-DIG antibody (1:8000, Roche) and stained with nitro blue tetrazolium (NBT) (Roche) and 5-bromo-4-chloro-3-indolyl phosphate (BCIP) (Roche) for the alkaline phosphatase reaction. 

### 4.11. Statistical Analysis

Values are presented as the mean ± s.e.m. and were compared by using Student’s *t*-test or one-way ANOVA. Statistical analysis was conducted using Prism 7.

## Figures and Tables

**Table 1:** Specific primer sequences of quantitative real-time polymerase chain reaction (qRT-PCR).

Gene Name	Protein Name	Forward Primer(5-3)	Reverse Primer(5-3)
*avp*	Avp	CGGAGCCCATCAGACAGT	TCGCAGCAGATGCCCTCA
*avpr1aa*	Avpr1aa	CTTCTACGGGCCGGACTTTC	CGGGCTGCTGAGGACTAAACT
*avpr1ab*	Avpr1ab	CGACTTCTTAGGCTGTTTCC	TAGGCACGCTCTGACTTGAT
*avpr2aa*	Avpr2aa	CCCGCAGATGTTATGGGATA	AGGCTACCATGATGGGTGTA
*avpr2ab*	Avpr2ab	TGTGACGAAAGCCATGTCTAAG	TGTGACGAAAGCCATGTCTAAG
*avpr2l*	Avpr2l	ATGGGCGCTCAAGCACTAAG	CCGTATGTCAGAGTGGCTTT
*rpl13a*	Rpl13a	CCTCGGTCGTCTTTCCGCTATTG	CAGCCTGACCCCTCTTGGTTTTG
*slc12a10.2*	Ncc2b	GCCCCCAAAGTTTTCCAGTT	GGCATGGAGCCTGTGATTG
*clc-2c*	Clc-2c	ATTGAGAAATGGGAGGAGCA	GGCATGCAGCCTGTGATG
*slc4a-4b*	Nbce1b	TGTTCCTCTACATGGGCGTCG	CAACCCACATAAATGATGACATC
*atp6v1a*	Ha	GAGGAACCACTGCCATTCCA	CAACCCACATAAATGATGACATC
*nhe3b*	Nhe3b	TGCAGACAGCGCCTCTAGC	TGTGGCCTGTCTCTGTTTGC
*slc4a1b*	Ae1b	GTCTGCGAAGAGCCCGAACC	CGGTGTTCATTGTCCTGCGTAT
*trpv6*	Ecac	TCCTTTCCCATCACCCTCT	GCACTGTGGCAACTTTCGT
*atp2b2*	Pmca2	AAGCAGTTCAGGGGTTTAC	CAGATCATTGCCTTGTATCA
*slc8a1b*	Ncx1b	TAAAGTGGCAGCGATACAGGT	CAGATCAAGGCGAAGATGG
*atp1a1a.1*	Nka.1	CATCCAGTCTGCATCACACAAG	TGGTTCACGATCTCAGTGTTTG
*cgrp*	Cgrp	CGACTACGAGGCGAGAAGATTG	CTCAGAAAGTCTGCCAGGCGAT
*crlr1*	Crlr1	AGCAGTGGCCAACAATCAAGA	CAAACACTGCCACAACAATGAG
